# Motivational Framing Strategies in Health Care Information Security Training: Randomized Controlled Trial

**DOI:** 10.2196/73245

**Published:** 2025-11-07

**Authors:** Thomas Keller, Julia Isabella Warwas, Julia Klein, Richard Henkenjohann, Manuel Trenz, Simon Thanh-Nam Trang

**Affiliations:** 1 Department of Business Education University of Hohenheim Stuttgart Germany; 2 Research Group on Information Security and Compliance University of Göttingen Göttingen Germany; 3 Chair of Interorganizational Information Systems University of Göttingen Göttingen Germany; 4 Chair of In­form­a­tion Sys­tems, in par­tic­u­lar Sus­tain­ab­il­ity Paderborn University Paderborn Germany

**Keywords:** cyber security, health care professionals, motivational framing, training programs, skill acquisition

## Abstract

**Background:**

Information security is a critical challenge in the digital age, especially for hospitals, which are prime targets for cyberattacks due to the monetary worth of sensitive medical data. Given the distinctive security risks faced by health care professionals, tailored Security Education, Training, and Awareness (SETA) programs are needed to increase both their ability and willingness to integrate security practices into their workflows.

**Objective:**

This study investigates the effectiveness of a video-based security training, which was customized for hospital settings and enriched with motivational framing strategies to build information security skills among health care professionals. The training stands out from conventional interventions in this context, particularly by incorporating a dual-motive model to differentiate between self- and other-oriented goals as stimuli for skill acquisition. The appeal to the professional values of responsible health care work, whether absent or present, facilitates a nuanced examination of differential framing effects on training outcomes.

**Methods:**

A randomized controlled trial was conducted with 130 health care professionals from 3 German university hospitals. Participants within 2 intervention groups received either a self-oriented framing (focused on personal data protection) or an other-oriented framing (focused on patient data protection) at the beginning of a security training video. A control group watched the same video without any framing. Skill assessments using situational judgment tests before and after the training served to evaluate skill growth in all 3 groups.

**Results:**

Members of the other-oriented intervention group, who were motivated to protect patients, exhibited the highest increase in security skills (ΔM=+1.13, 95% CI 0.82-1.45), outperforming both the self-oriented intervention group (ΔM=+0.55, 95% CI 0.24-0.86; *P*=.04) and the control group (ΔM=+0.40, 95% CI 0.10-0.70; *P*=.004). Conversely, the self-oriented framing of the training content, which placed emphasis on personal privacy, did not yield significantly greater improvements in security skills over the control group (mean difference=+0.15, 95% CI –0.69 to 0.38; *P*>.99). Further exploratory analyses suggest that the other-oriented framing was particularly impactful among participants who often interact with patients personally, indicating that a higher frequency of direct patient contact may increase receptiveness to this framing strategy.

**Conclusions:**

This study underscores the importance of aligning SETA programs with the professional values of target groups, in addition to adapting these programs to specific contexts of professional action. In the investigated hospital setting, a motivational framing that resonates with health care professionals’ sense of responsibility for patient safety has proven to be effective in promoting skill growth. The findings offer a pragmatic pathway with a theoretical foundation for implementing beneficial motivational framing strategies in SETA programs within the health care sector.

## Introduction

### Background

In the digital era, information security has emerged as a pivotal challenge for a wide array of industries, most notably for critical infrastructure, where interruptions can exert detrimental impacts on public safety, the economy, and the daily lives of individuals [1]. Hospitals have become a primary target for cyberattacks due to the substantial financial value of sensitive medical data [2]. The *Ponemon Healthcare Cybersecurity Report 2023* indicates that 88% of health care organizations experienced an average of 40 cyberattacks in the past 12 months, significantly impacting patient care and causing financial losses amounting to millions of dollars [3]. The prevalence of incidents underscores the need to enhance measures of information security protection [4]. However, technological vulnerabilities are not the sole source of threat. The human factor frequently serves as a crucial gateway for criminals. Errors, negligence, and inadequate knowledge about countermeasures all contribute to successful cyberattacks [5]. In particular, a lack of security awareness encourages noncompliance with organizational security policies, thus leading to risky workplace behaviors such as using weak passwords and unsecured devices [6]. As a consequence, hospital staff unknowingly create vulnerabilities that can be exploited by attackers [7]. This problem is exacerbated by hospitals' increasing reliance on interconnected digital systems [2,8], including IoT-based health care solutions [9], and the high-pressure environment of health care work, which often prioritizes rapid patient care over rigorous security measures [10].

In response, health care organizations are expanding the implementation of Security Education, Training, and Awareness (SETA) programs to improve their employees’ information security skills [11]. Despite these efforts, many SETA programs have proven ineffective in fostering secure working practices over the long term by focusing on formal regulatory requirements without addressing the work processes of health care professionals and, thus, the application contexts of users [12-15]. Adapting SETA programs to the tasks and inherent security threats that health care professionals face every day seems imperative [16]. However, this target group rarely perceives information security as part of their primary job responsibilities [17]. Despite the provision of domain- and context-specific knowledge through training, health care professionals may still rate the importance of safeguarding medical data in their daily workflows as low [18]. A substantial proportion of professionals even demonstrate a flawed understanding of the consequences of inadequate security practices, including ransomware attacks that access electronic health records or data manipulations that lead to treatment errors [5]. This inner devaluation of risky behaviors and their consequences threatens the confidentiality, availability, and integrity of sensitive patient data. In essence, motivational deficits on the part of the professionals can be a serious barrier for acquiring skills that are necessary to act in accordance with security policies at work—even if training content is adequately customized to match their daily professional activities.

### Enhancing Training Engagement Through Motivational Framing Strategies

A recognized method of invigorating involvement in training and promoting compliance with information security policies at work applies *fear appeals*, which raise awareness of the *personal relevance* of counteracting security threats [19,20]. As Johnston et al [21] point out with reference to protection motivation theory (PMT), powerful fear appeals address both formal sanctions (eg, punishment and loss of valuable information) and informal consequences (eg, social disapproval and reputational damage) in future cases of noncompliance. Nevertheless, an exclusive emphasis on avoiding unfavorable outcomes may not be the most effective strategy to encourage engagement with training content, as evident in research on controlled versus autonomous motivation of learning [22]. Research based on self-determination theory [23] shows that controlled motivation, such as compliance driven by external pressure, often leads to superficial learning. By contrast, training approaches that cultivate autonomous motivation by connecting to individual goals or professional purpose are more likely to elicit deeper engagement and enduring behavioral change [24]. Moreover, where consequences for unnoticed violations are missing, there may be little long-term motivation to follow information security rules in daily work [19].

*Motivational framing* offers a promising alternative for establishing the personal relevance of secure workplace behaviors and encouraging their acceptance as an integral part of one’s professional practice. It aligns the content and structure of a message or topic with the motivational states, pursued goals, or cherished values of the target audience, thereby increasing the topic’s subjectively assessed importance [25]. Joyal-Desmarais et al [26] report that motivational framing has a strong impact when the message aligns specifically with the individual's intrinsic motivation or core values. Their meta-analysis of 702 experimental studies highlights how messages that are tailored to resonate with such personal reference points of the recipients can significantly increase the persuasiveness of provided information, thus contributing to marked changes in attitudes, intentions, and behaviors. This raises the question of which core values a security training in hospitals should best appeal to.

Since hospital staff provide assistance to others, their professional activities can most generally be termed prosocial [27]. According to the *dual-motives model,* however, prosocial behavior can be driven either by self-serving (self-oriented) or by altruistic (other-oriented) motives [28]. While the former focus on personal benefits, such as enhancing one’s reputation, gaining recognition, or fulfilling job-specific duties as part of one’s paid employment, the latter prioritize the needs and well-being of others, such as ensuring the safety and dignity of patients [29]. A closer investigation of the *core professional values* endorsed by health care professionals strongly suggests that an other-oriented (ie, patient-oriented) framing of training content on information security could indeed function as the most effective prompt to encourage attention and engagement—an assumption that will be further elaborated in subsequent sections.

At this point, we can conclude that the “personal relevance” of averting security threats as a health care professional could become more of a voluntary activity in accordance with one's professional values than merely a pointless duty imposed by regulatory bodies if training participants are prompted to detect this accordance. The general idea of addressing a professional commitment as a motivational stimulus is also fully compatible with the Fogg Behavior Model (FBM). The FBM posits that the performance of a target behavior is contingent upon 3 factors: sufficient motivation, the capacity to execute this behavior, and an adept cue that prompts its execution. The occurrence of these factors must be simultaneous for the behavior to manifest [30]. A training program that incorporates appeals to those salient values that generally drive professional action and, thus, enriches practical applicability with targeted messages or cues, therefore, seems particularly adept for building individual capacities for secure workplace behaviors.

Against this background, this study seeks to determine whether a motivational framing of information security training content, which varies in its alignment with the professional values of the target audience, impacts the acquisition of information security skills to varying degrees.

### Hypotheses Development

The training for health care professionals has been meticulously developed, integrating problem-solving approaches to learning and recommended features for video formats while delivering domain-specific, contextualized content (see section on Instructional and Assessment Design for details). Therefore, the investigated hypotheses focus on the “dual” strategies of motivational framing and their potential to establish a connection between the adoption of information security practices and the commitment to professional values.

Since core professional values form the foundation of health care practices and the inner ethical obligations of health care staff [31], appeals to these values should promote engagement with learning material in an effort to expand one's repertoire of security skills [32]. Among these values, patient-centered care stands out as a primary commitment, emphasizing the safety, dignity, and well-being of patients. Professional integrity, another critical value, entails adherence to ethical standards and accountability, particularly in safeguarding sensitive patient information. Altruism and compassion further underscore the motivation of health care professionals to prioritize patient needs, often going beyond contractual obligations to act in the best interest of those they serve [33]. Consequently, an *other-oriented* framing of training content, which directs the participants toward *protecting information of and about their patients,* should be most adept to foster the acquisition of information security skills. In support of this idea, White and Peloza [34] report that people are more likely to respond to other-oriented appeals in contexts where social responsibility is emphasized.

Nevertheless, self-oriented appeals, emphasizing the individual benefits of protecting personalized data, should not be without any effects. Support for this perspective comes from PMT [35], which argues that individuals are most likely to engage in protective behavior when they anticipate being affected individually by the consequences of successful threat aversion or their failure to do so, while concurrently believing that they possess the knowledge and tools to respond adequately. Self-oriented framing consequently stresses these personal risks and the importance of self-protection. It makes consequences, such as service disruption or privacy loss, more immediate. Research supports the notion that perceived personal vulnerability drives individuals to adhere to safety measures. AlSobeh and colleagues' [36] findings suggest that adolescents who perceive cybersecurity risks to impact their personal quality of life demonstrated a heightened level of security awareness. In accordance with this line of thinking, self-oriented framing is appealing to defenses of one's own (informational) integrity.

Therefore, we expect an other-oriented framing of information security training, accentuating patient protection, to promote greater skill acquisition than delivering the training without any framing. It should also be superior to self-protection appeals, which should again be more effective than no framing at all. This leads to the following hypotheses:

- Hypothesis 1: Participants who receive a self-oriented framing of training content will demonstrate higher levels of skill acquisition compared to a control group without any framing.
- Hypothesis 2: Participants who receive an other- (ie, patient-) oriented framing will demonstrate higher levels of skill acquisition compared to those with a self-oriented framing.

## Methods

### Instructional and Assessment Design

#### Enhancing Information Security Skills Through Job-Specific, Problem-Oriented, Video-Based Training

In determining the relevant skillset to be acquired, we drew on a framework that was developed specifically for information-secure workplace behaviors [37,38]. The model proposes professional activity to be targeted, knowledgeable, justifiable, and responsible [39,40]. It further aligns with models of (expert) problem-solving [41,42] by positing that a proficient handling of security threats spans a comprehensive cycle of reasoned action. This cycle starts with an early detection of risky situations within one's own working environment and extends up to the execution of follow-up procedures that are beneficial for the whole team or organization. Thus, the skillset comprises seven elements. (1) *Threat awareness* emphasizes the ability to recognize potential security threats and remain vigilant, distinguishing between threatening and nonthreatening work situations. (2) *Threat identification* focuses on accurately identifying the presence of a security threat and understanding its specific nature. (3) *Threat impact assessment* ensures that individuals understand the consequences of not addressing current security threats. (4) *Tactic choice* enables individuals to select appropriate measures by aligning their actions with prevailing best practices and established rules of information security while tailoring those actions to the specific threat scenario they face. (5) *Tactic justification* emphasizes the importance of a structured, goal-oriented approach in which individuals can justify their actions based on relevance, effectiveness, and superiority. With (6) *tactic mastery,* individuals can effectively implement the security measure of choice. Finally, (7) *tactic check and follow-up* involves evaluating the effectiveness of implemented measures and, where possible, taking follow-up actions to further improve the organization's security.

A critical evaluation of the strengths and limitations of various training methods (for an overview, see [43]) revealed that video-based training offers a promising solution for the health care sector. First, it provides a largely visual presentation of content, thereby facilitating the acquisition of knowledge and skills regardless of the learners’ literacy [44,45]. Second, health care professionals often have unpredictable schedules and limited time for instructor-led classroom training. In such circumstances, training videos provide a convenient and accessible learning route, ensuring the delivery of essential strategies and reasoning in a clear, concise, and engaging manner [46]. Third, the standardization and repeatability of video-based training material fosters the dissemination of consistent, high-quality information to all employees, enabling them to progress through the material at their own pace [47].

**Figure 1:**
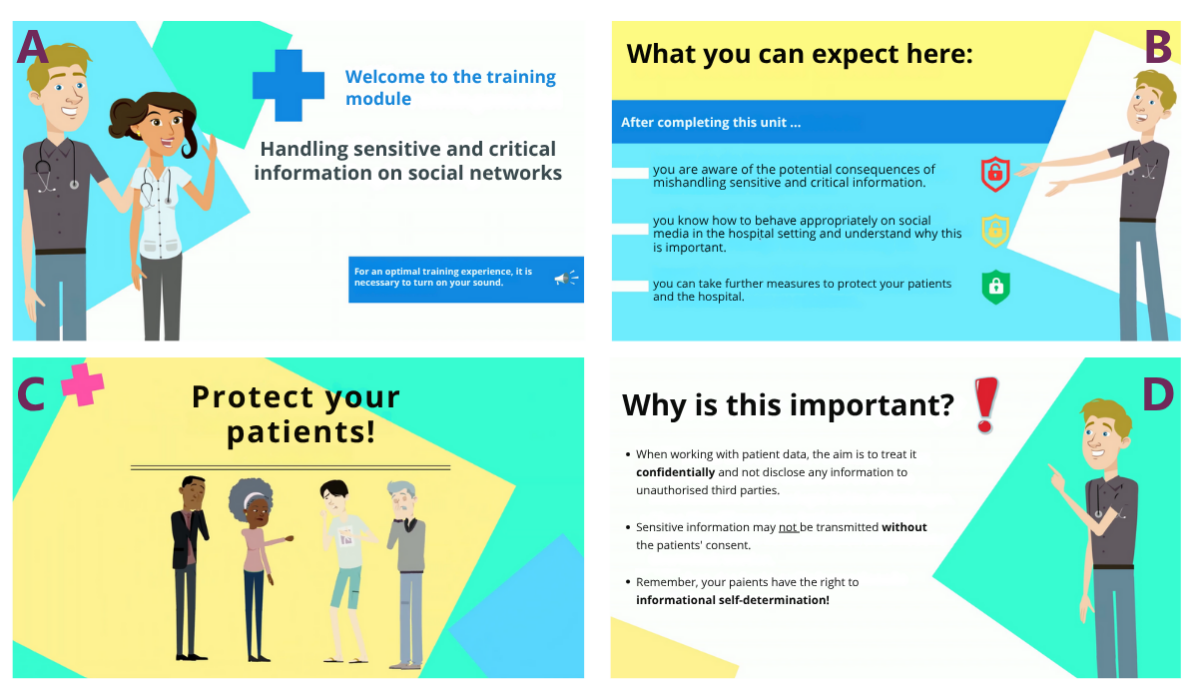
Design highlights from the training module "handling sensitive and critical information on social networks.”.

Several research-based recommendations were implemented in the video production. Given the condensed information typically conveyed in this format, empirical studies suggest limiting the duration of training videos to no more than 6 minutes to maintain attention and engagement [48,49]. Storytelling and sensory activation [50,51] appeal to different learning styles [52] and promote information retention. At the beginning of each training video, participants are presented with general orienting information, including the learning objectives to be achieved, the approximate completion time, and an explanation of the badges to be earned [53]. These badges provide transparency of individual learning progression (beginner, advanced, or expert), thereby contributing to learner motivation and self-directed learning [54]. Upon completion, participants have mastered all training content for analyzing and averting security threats. Figure 1 illustrates these design principles as a multipanel overview covering the key visual elements of the training: (A) introduction, (B) learning objectives and badges, (C) other-oriented nudge, and (D) the “tactic justification” dimension.

In line with the general objective of SETA programs described in the introduction, the training aimed to develop the ability to select and implement safe behaviors that are compatible with the respective employee's daily work and that are particularly relevant to the risk factors present there. Based on a comprehensive analysis of information security behavior in the health care sector [16], different *threat vectors* have been identified for typical *job profiles*. These job profiles differ in their levels of interaction with patients and their reliance on IT systems, which directly influence their exposure to information security risks.

Physicians interact with patients frequently and use IT systems moderately to extensively, depending on their responsibilities for diagnosis, treatment planning, and documentation. Common threats they face include the *inadvertent disclosure* of sensitive information, for example, by forwarding patient-related data via private messaging services, as well as *spear phishing*, where attackers send personalized emails designed to appear legitimate.

Nurses typically have the most frequent patient contact but rely on IT systems only to a limited extent, mainly for tasks such as documentation and medication administration. They, too, are particularly vulnerable to *inadvertent disclosure* of sensitive information, such as through social networking or the use of private messaging services, which could be used for expeditious communication.

Administrative staff rarely interact directly with patients but rely heavily on IT systems. Tasked with the critical function of managing both digital and physical (patient) data, scheduling, and billing, they are frequent targets of *phishing attacks,* such as fake websites. Additionally, a disorganized workspace increases the probability of sensitive information being exposed on desks or computer screens. Failure to comply with the hospital’s *clean desk policy* thus increases their risk of granting unauthorized access and violating data confidentiality.

By assessing the criticality and phenomenology of these threat vectors through expert interviews [16], we designed customized training videos that specifically address these prevalent and consequential security threats of different job profiles. In line with the problem-solving skills model described above, the videos take the occurrence of threat vectors as the starting point of a problem-solving process in a prototypical workplace scene. Thus, each video begins with visual or auditory stimuli that represent variants of an authentic workplace situation that could pose an information security threat, encouraging employees to assess associated risks and participate in finding a solution [55].

#### Creating Authentic Learning Assessments Through Situational Judgment Tests

Situational judgment tests (SJTs) provide a robust approach to assessing general and job-specific procedural knowledge in an authentic environment. They are applicable to a wide range of fields, including personnel selection and development, medical licensing and certification, education, and psychological assessment (eg, personality) [56-58]. Moreover, they hold significant potential for expanding into emerging applications, including health behavior and interpersonal skills [59]. SJTs are characterized by a strong intuitive prompt that asks test takers to place themselves in specific situations [60]. In this study, participants face a security threat scenario. Their task is then to select the most adept judgments and the most effective actions from several alternative responses, which are presented hereafter. In accord with the problem-solving cycle delineated above, which guided the participants through the training videos, the test items for evaluating their skill gains cover 7 dimensions, ranging from *threat awareness* to *tactic check and follow-up*. Each of these dimensions was represented by one test item within a thematically coherent testlet based on a realistic threat scenario.

**Figure 2:**
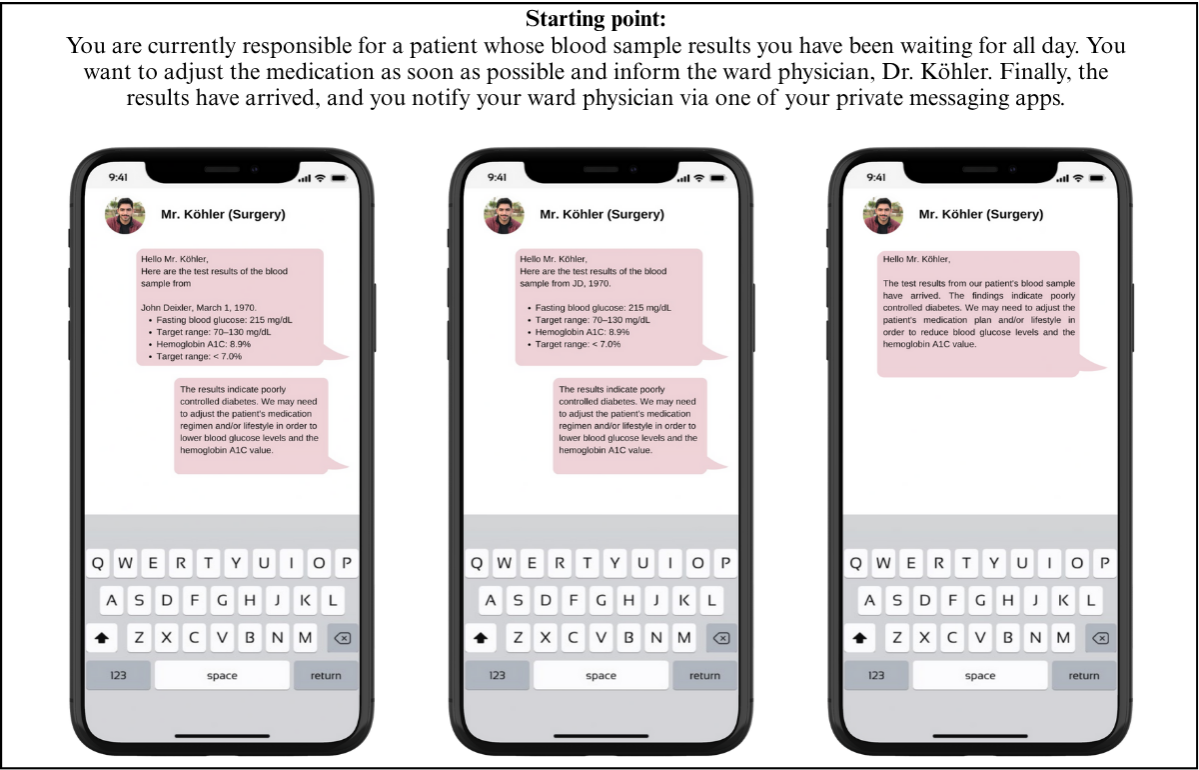
Example item from the situational judgment test targeting the dimension “threat awareness.”.

Thus, the central tenets of the developed SJTs lie in their contextualization and authenticity. By immersing participants in a fictional university hospital and presenting prototypical threat scenarios through authentic image and video stimuli, these SJTs provide a cost-effective and valid method to assess employees' capabilities to manage these threats without the need to simulate factual security incidents. This approach is particularly salient in professional contexts where interruptions pose a significant threat to patient health and, in extreme cases, patient lives. The impracticality, cost, and ethical challenges associated with extensive simulations of, for example, data breaches render SJTs a compelling alternative. An example of such a scenario is shown in Figure 2, illustrating the *threat awareness* dimension of the SJT. Participants were asked to evaluate the relative severity of various messaging behaviors that could lead to the inadvertent disclosure of sensitive patient data via private messaging apps, and to rank them using the following scale: 1=most threatening, 2=less threatening, and 3=least threatening.

In several workshops, we not only developed problem-based threat scenarios but also professionally grounded response options for information-secure actions. The scenarios and response options were iteratively revised in close collaboration with information security experts and test developers, who paid particular attention to their alignment with the respective skill dimensions and security policies. To allow for a differentiated assessment of both high and low levels of the targeted construct, the number of response options per item was limited to 4 to 6. The tests used a forced response format with different response types, including single choice, multiple choice, and rating, to enable a nuanced assessment of decision-making and judgment in information security contexts. To examine the quality of the testlets, we conducted a pretest with 100 physicians (37 female, 63 male; mean age 34.73, SD 10.95 years), 101 nurses (41 female, 60 male; mean age 41.40, SD 9.85 years), and 102 administrative staff (24 female, 76 male, 2 diverse; mean age 28.26, SD 7.29 years). The results indicate an acceptable level of difficulty for the target population, as reflected in the range of the item difficulty index (0.71-0.78). In addition, key metrics such as variance and discrimination index confirmed that the test items effectively detect interindividual differences in test performance. Consistently positive feedback on the usability and authenticity of the test underscored its acceptance among the target groups [37].

### Intervention Implementation

**Figure 3:**
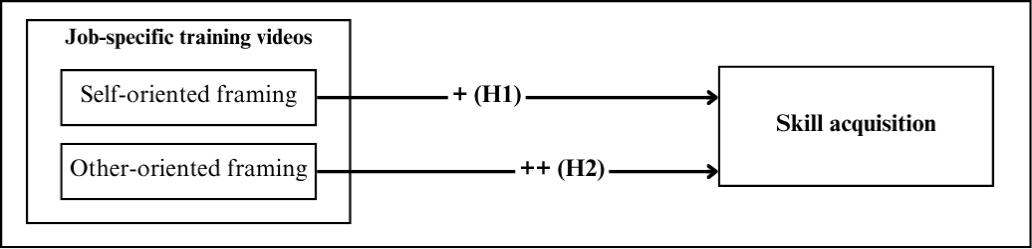
Conceptual model illustrating the hypothesized effects of self-oriented and other-oriented motivational framing of job-specific training videos on skill acquisition.

To test our hypotheses and proposed research model (Figure 3), we conducted a randomized controlled trial with hospital staff of 3 German university hospitals in late 2023. Data were collected via the online platform Qualtrics. The participating organizations were free to decide whether to share the link to this platform in their intranet or via newsletter, sent from either their marketing or their information security department. The training phase was open for 2 weeks, during which time participants could schedule the training at their convenience. At the beginning, participants were asked to indicate their job profile. Based on this information, participants were assigned a job-specific training video and corresponding pre- and posttests, each tailored to the demands and risks associated with their professional role. An attention check in the form of a single content-related question was included after the video, without revealing or overlapping with any later test content. All participants correctly answered the attention check question, indicating continuous attention to the video content. Additionally, the time participants spent on the training page was recorded as an indicator of intervention exposure. To ensure standardization and minimize external influences, all participants completed the baseline-skill assessment (pretest), the video intervention with or without motivational framing, and the achieved-skill assessment (posttest) in immediate succession on the platform.

### Randomization and Blinding

Within each job profile, participants were randomly assigned to either the treatment or control condition. The allocation was conducted using simple randomization through the online platform at the start of the study. This automated process was embedded in the predefined succession of tests and training (see above) to ensure allocation concealment and prevent any influence from the researchers or participants on group assignment. Professionals in the control group were provided only with their respective job-specific training videos. Those in the two intervention groups watched the same videos (for their profile) with an additional motivational framing at the beginning that was either self-oriented or other-oriented (Table 1). The total length of the training videos was approximately 5 minutes of pure information security content and an additional 30 seconds for a motivational framing. Participants were unaware that different motivational framings were used in the training materials and assumed that all participants received the same intervention. Skill assessment in both the pre- and the posttests was fully automated through a predefined scoring algorithm. No manual input or subjectively biased coding influenced the outcome data, ensuring objectivity and consistency.

**Table 1:** Overview of motivational framing strategies in the intervention groups.

Group	Motivational framing
Other-oriented framing (n=43)	The dangers posed by incorrect IT security behavior are very high and can lead to the interruption of services or the disclosure of sensitive data. Therefore, it is important that you are able to protect your patients. The content from this training will help you build the skills necessary to protect your patients and their privacy.
Self-oriented framing (n=43)	The dangers posed by incorrect IT security behavior are very high and can lead to the interruption of services or the disclosure of sensitive data. Therefore, it is important that you are able to protect yourself. The content from this video will help you build the skills necessary to protect yourself and your privacy.
Control (n=44)	No motivational framing.

### Measures

The intervention's efficacy was evaluated using SJTs, which were developed to assess information security skills. To serve the purpose of pre- and posttraining assessments, these SJTs used 2 threat vectors from the same risk category for each job profile. For physicians and nurses, the risk category of inadvertently disclosing sensitive information was operationalized in two ways: (1) disclosing sensitive patient data via private messenger services and (2) publishing images via social networks. For administrative staff, two threat vectors were developed to reflect noncompliance with the clean desk policy: (1) leaving confidential documents openly visible on the desk and (2) leaving sensitive electronic data accessible by failing to lock the computer when unattended. Therefore, the pre- and posttest scenarios and their related items were not identical, although they followed the same structure and belonged to the same risk category. Nonetheless, the SJTs were presented in a randomized order to minimize potential biases arising from test familiarity. Each participant was randomly assigned 1 of the 2 SJTs corresponding to their job profile for the baseline assessment. The other test served to measure posttraining skill levels. Each test item, corresponding to 1 of the 7 information security skill dimensions, was scored from 0 to a maximum of 2 points, resulting in a total score of up to 14 points per testlet. Age and gender were included as control variables to account for potential demographic influences on skill gains.

### Participants

A total of 130 participants were included in the study, with a balanced gender distribution (53.8% male and 46.2% female). Their age ranged from 18 to 71 years (mean 42.6, SD 12.21 years). Regarding job profiles within the health care sector, 50% of the participants primarily fulfilled administrative tasks in their respective hospitals, 30.8% were occupied as nurses, and another 19.2% as physicians. This diverse sample composition allows for a comprehensive analysis of the effects of the intervention across different job profiles.

### Power Calculation

A power analysis was conducted using the R package pwr [61] to determine the necessary sample size for a 1-way ANOVA with 3 groups. Assuming a medium effect size (*f*=0.30 [62]), an alpha level of .05, and a desired statistical power of 0.80, at least 37 participants would be needed per group, which was exceeded in the present sample. The underlying assumption was supported by effect sizes reported in comparable studies on motivational framing and message matching, which demonstrated moderate effects on attitude and behavior change [26].

### Data Analysis

Before running the main analysis, we compared the baseline characteristics of the experimental groups to ensure group equivalence at the beginning of the study. Categorical variables were analyzed using chi-square tests, while age was examined using 1-way ANOVA. Baseline skill levels (pretest scores) were compared using Kruskal-Wallis tests to account for potential violations of normality. No significant differences were found between groups with respect to age (*P*=.39), gender (*P*=.95), or job profile (*P*=.91). However, baseline skill levels differed significantly between groups, as indicated by the Kruskal-Wallis test (χ²_2_=9.4, *P*=.009). Therefore, the baseline skill level was included as a covariate in subsequent analytical steps. This allowed us to adjust for initial differences in participants’ prior knowledge and to isolate the effect of motivational framing on skill gain with greater precision.

Before testing hypotheses of motivational framing, exploratory paired-samples *t* tests within each job profile were conducted. This step established the training intervention's baseline efficacy, allowing the interpretation of any estimated framing effects as modulators of learning processes. Its outcome is reported in the descriptive results below.

Building on this, we examined the expected differential extents of skill acquisition yielded by the applied types of motivational framing. An analysis of covariance (ANCOVA) was performed with skill acquisition (measured by the difference between posttest and pretest scores) serving as the dependent variable and the experimental condition (control, self-oriented, or other-oriented) representing the independent variable. The requirements for applying ANCOVA are fulfilled. Observations were independent by design, as each participant was assigned to only one experimental condition. The assumption of homogeneity of variances was met, as indicated by the Levene test (*F*_2,127_=0.37; *P*=.69). To detect potential outliers in skill acquisition within each experimental group, we calculated *z* scores separately for each group. No outliers were identified based on this criterion, suggesting that extreme values in skill acquisition were not present within any experimental condition. Although the Shapiro-Wilk test (W=0.967; *P*=.003) indicated a slight deviation from normality, given the sample size and the demonstrable absence of extreme outliers, the ANCOVA can be considered to deliver robust estimations [63]. The assumption of homogeneity of regression slopes was also fulfilled, as the interaction between group and pretest score was not significant (*F*_2,124_=0.30; *P*=.74). All statistical analyses were conducted using R version 4.3.2 (October 31, 2023; R Core Team).

### Ethical Considerations

The study was reviewed by the ethics committee of the Georg-August University of Göttingen, Germany (approval issued May 8, 2023). In an official statement issued in May 2023, the committee raised no ethical concerns about implementing the project as long as all applicable data protection regulations were followed. All participants were informed of the purpose of the training and how the tests would be used to evaluate its effectiveness. Participation was voluntary, and informed consent was obtained beforehand. Data handling complied with the General Data Protection Regulation and institutional data protection policies. No financial or material compensation was provided to participants.

## Results

### Descriptive Results

**Figure 4:**
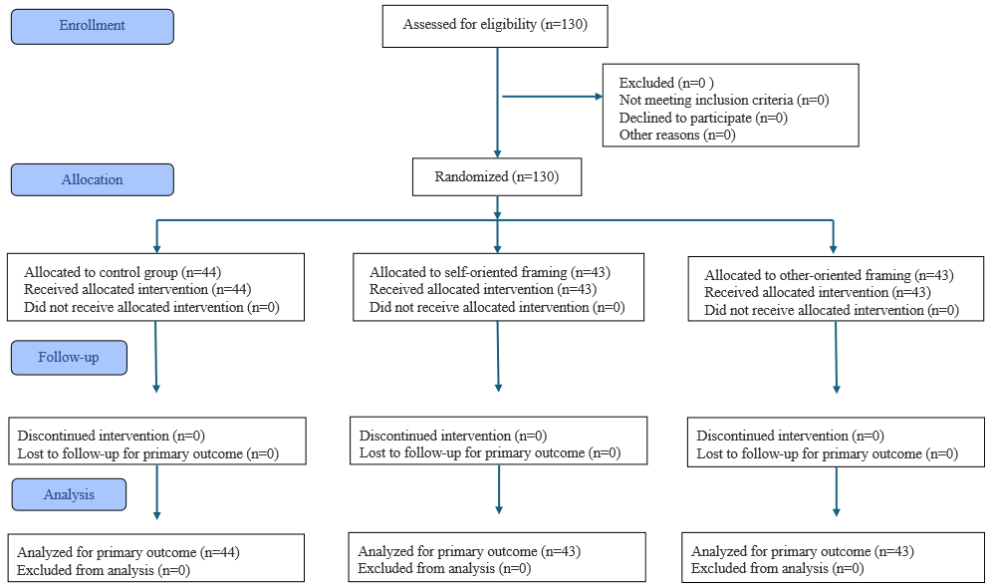
CONSORT flow diagram illustrating the progression of participants through the randomized controlled trial. CONSORT: Consolidated Standards of Reporting Trials.

Building on the sample description above, Figure 4 depicts the flow of participants through recruitment, allocation, and analysis in accordance with the CONSORT (Consolidated Standards of Reporting Trials) guidelines [64] (checklist in Multimedia Appendix 1). Table 2 shows the descriptive study results for the 3 job profiles sorted by the experimental groups. Paired-samples *t* tests (2-tailed) indicate significant gains in information security skills in all job profiles following the training intervention. For administrative staff, the analysis reveals a significant improvement from pretest scores to posttest scores (*t*_64_=3.30; *P*=.002) with a mean difference of 0.51 (95% CI 0.20-0.81) and an effect size of Cohen *d*=0.41. Similarly, a significant increase was observed for nurses (*t*_39_=2.63; *P*=.01), with a mean difference of 0.65 (95% CI 0.15-1.15) and an effect size of *d*=0.48. The greatest improvement was found among physicians (*t*_24_=4.11; *P*<.001), with a mean increase of 1.24 (95% CI 0.62-1.86) and an effect size of *d*=1.21. Taking an average across all job profiles, information security skills reached significantly higher levels after the training compared to before the training, with a mean difference of 0.69 (95% CI 0.45-0.94; *t*_129_=5.58; *P*<.001), and an overall effect size of *d*=0.52, indicating a medium effect. These findings document that skill gains were not limited to an average value for the entire sample, but were consistently observed within each job profile.

**Table 2:** Pretest, posttest, and skill acquisition scores by experimental group and job profile.

Intervention condition and job profile		Control group (n=44), mean (SD)	Self-oriented framing (n=43), mean (SD)	Other-oriented framing (n=43), mean (SD)
**Administrative staff (n=65)**				
	Pretest	12.42 (1.25)	12.81 (1.33)	12.05 (1.28)
	Posttest	12.67 (1.20)	13.00 (1.22)	13.20 (1.06)
	Skill acquisition	+0.25 (1.33)	+0.19 (1.12)	+1.15 (1.04)
**Nurses (n=40)**				
	Pretest	10.92 (1.66)	12.07 (1.27)	10.77 (1.54)
	Posttest	11.54 (1.13)	12.07 (1.07)	12.15 (0.99)
	Skill acquisition	+0.62 (1.66)	0.00 (1.36)	+1.38 (1.45)
**Physicians (n=25)**				
	Pretest	12.86 (0.69)	12.13 (1.13)	11.20 (1.32)
	Posttest	13.14 (0.69)	13.13 (0.83)	13.30 (0.67)
	Skill acquisition	+0.29 (0.76)	+1.00 (1.31)	+2.10 (1.66)

### Impact of Motivational Framing Strategies on Skill Acquisition

While overall improvements in information security skills are evident, the question remains whether the specific type of motivational framing influenced their magnitude. To address this, we tested hypotheses 1 and 2 using an ANCOVA procedure. The results reveal a significant main effect of motivational framing on skill acquisition, controlling for initial skill level (*F*_2,126_=5.92; *P*=.004; partial η²=0.09). Post hoc pairwise comparisons with Bonferroni adjustment across all job profiles indicate that the other-oriented framing group achieved significantly greater skill growth (mean +1.133, SD 0.159) compared to the control group (mean +0.400, SD 0.154; *t*_126_=3.30; *P*=.004), as well as to the self-oriented framing group (mean +0.551, SD 0.158; *t*_126_=2.54; *P*=.04). No significant differences were found between the control and self-oriented framing groups (*t*_126_=0.687, *P*>.99). In addition to the effect of the framing strategy, a substantial portion of the variance in skill growth can be attributed to each participant’s baseline skills (*F*_1,126_=86.88; *P*<.001; partial η²=0.41). Participants who started at a higher skill level demonstrated smaller gains from the training videos than those who started at lower or even rudimentary skill levels. These findings offer substantial evidence for hypothesis 2, which predicted the other-oriented framing of training content to be superior to a self-oriented framing in the studied target audience of health care professionals. Contradicting hypothesis 1, however, self-oriented framing did not yield greater enhancements of information security skills than no framing at all.

Furthermore, a closer inspection of the descriptive means presented in Table 2 suggests that the generally superior effectiveness of the other-oriented framing (see hypothesis 2 above, comparing intervention and control groups) appears to resonate more strongly with certain job profiles than with others. Physicians demonstrated the largest improvement (mean increase +2.10), followed by nurses (mean increase +1.38), and administrative staff (mean increase +1.15), whose gains, although statistically significant, were markedly smaller. This variability suggests that although all professional sectors within hospitals responded to an other-oriented motivational framing of training content, the extent of responsiveness may be influenced by individual or contextual characteristics. Drawing on models of social responsibility and professional identity [31,32], the degree of personal and direct patient contact may influence how strongly hospital staff respond to other-oriented appeals. Specifically, the frequency with which one engages personally and directly in medical and care work, as well as ethically charged decision-making, may further increase the salience of other-oriented appeals, thereby enhancing their persuasive potency.

A rigorous moderation analysis examining other-oriented framing effects contingent on contact frequency was not feasible, as a direct and isolated measure of individual patient contact was unavailable in this study. However, contact frequency is typically, although not deterministically, higher for certain job profiles than for others (see profile descriptions above). Consequently, we grouped nurses and physicians to compare them to the administrative staff. Nurses and physicians are embedded in the core clinical care process. They operate in close proximity to patients and often form interprofessional treatment teams within inpatient settings. Both focus on health-related goals and are most immediately responsible for the well-being of vulnerable individuals. Administrative staff primarily perform organizational and procedural tasks that serve patients' best interests, such as ensuring they receive swift treatment and proper accommodations. However, most of these tasks are structurally removed from the clinical care environment.

**Figure 5:**
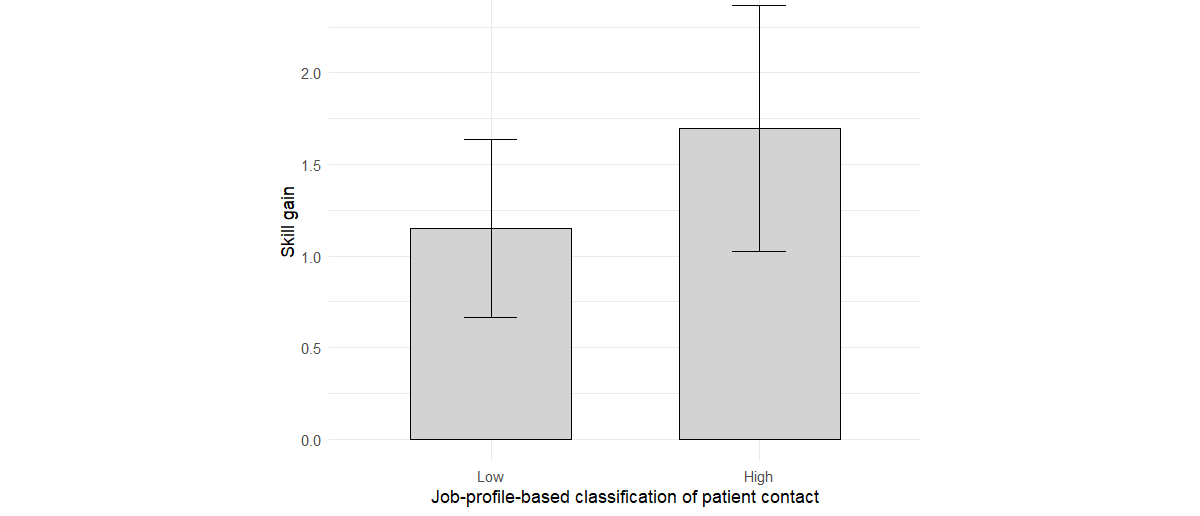
Mean skill gain by the "profile-typical" frequency of patient contact within the other-oriented framing condition.

Therefore, we conducted a descriptive subgroup analysis restricted to participants who received the other-oriented framing. Specifically, we compared skill gains between roles involving high (n=23) and low (n=20) frequencies of direct patient contact. We applied the Welch *t* test, which yields robust estimates. While the high-contact group showed greater skill gains (mean increase 1.70, SD 0.150) than the low-contact group (mean increase 1.15, SD 0.104), this difference did not reach statistical significance, *t*_38.67_=1.37, *P*=.18, 95% CI –1.35 to 0.26. Figure 5 illustrates this descriptive pattern, showing that when receiving training content that is framed toward patient protection, participants whose professional roles typically involve frequent direct patient contact experience an even stronger increase in skill gain than those in lower contact-frequency roles. However, this observation is limited to the present sample.

## Discussion

### Principal Findings

This study sheds new light on the effectiveness of motivational framing strategies in information security training for health care professionals. Participants of a video-based, job-specific training demonstrated significant improvements in information security skills. These improvements were observed across all job profiles, including administrative staff, nurses, and physicians. While empirical evidence that a contextualized and psychologically grounded content increases the effectiveness of security training has already been provided by previous studies [65], our randomized controlled trial with 130 participants demonstrates that the targeted activation of professional values can further enhance skill acquisition.

In particular, our results underscore the widely recognized limitations of traditional SETA programs, which focus on formal content and guidelines without addressing motivational aspects [66]. In this study, both the control group and the group that received a self-oriented motivational framing achieved comparably small skill gains. This indicates that appeals to individual protection or self-interest do not prompt heightened engagement with training content on information-secure workplace behaviors in professions with high levels of social responsibility, even if this content is well tailored to their daily tasks. Therefore, hypothesis 1 was not supported. This finding contrasts with studies from other professional sectors that used self-oriented *fear* appeals to promote secure behavior [67,68]. While self-oriented motivational framings have proven effective in private settings, such as protecting personal data, they appear to be less effective in clinical settings where the exertion of professional roles is strongly guided by professional-ethical values. As set out in the introduction, patient-centered care represents a primary commitment of health care professionals, which prioritizes the safety, dignity, and well-being of patients. Such concepts are integral to professional identity in the health care sector [33].

Consequently, and in accordance with hypothesis 2, framing security training content in an other-oriented way, thereby highlighting the protection of patients through information-secure workplace behaviors, proved to be the superior strategy. Participants in this intervention group demonstrated significantly greater improvements in information security skills compared to those in the control and self-oriented intervention groups. These results underscore the importance of aligning the messages that precede and accompany a training program with the core professional values of the target audience. The principle of value congruence [69], which posits that individuals respond more positively to messages that resonate with their core values, further substantiates this interpretation. Similar effects have been demonstrated in prosocial messaging research, where other-oriented appeals have a markedly stronger impact than self-oriented ones when applied in morally salient contexts [34]. Our findings extend this body of work to cybersecurity education, suggesting that value congruence can increase the effectiveness of SETA programs in socially responsible professions.

Supplementary exploratory examinations of subgroups indicate that certain variations in individual skill gains within the (generally superior) other-oriented framing condition might depend on the frequency of patient interaction. Participants whose job profiles typically require frequent direct and personal contact with patients, that is, physicians and nurses, displayed even greater increases in information security skills when the training content was framed in an other-oriented manner than administrative staff participants did. This (merely descriptive) pattern is consistent with identity-based motivation theory [70], which posits that individuals are more likely to respond to messages that activate their specific role characteristics in a social or professional community. Thus, it provides initial support for the idea that the frequency of patient interactions (as an individual feature of participants) may further enhance the general responsiveness of health care professionals to other-oriented approaches.

Taken together, the findings highlight the importance of value-based design in SETA programs. Aligning training content with the core professional values of health care workers, such as patient protection and social responsibility, can help interventions achieve better learning outcomes. This interdisciplinary approach is a promising avenue for future research and practice on cybersecurity education.

### Practical Implications

Not only the obtained empirical results but also the applied instructional and assessment design provide practical implications for the construction of SETA training and the implementation of motivational framing strategies. Structuring information security content around a substantiated skill model and tailoring it to employees' specific tasks and demands can arguably promote attention and engagement levels throughout the training. Put simply, professionals are more encouraged to extend their skill repertoire when the content is directly applicable to their daily work routines. Furthermore, tailored training programs are effective in reducing the duration of the program by eliminating irrelevant information. This approach prevents participants from feeling either overwhelmed by abstract input or overlooked as an agentic professional. Moreover, it facilitates an efficient use of training resources.

Explaining the repercussions and rationales for security-compliant conduct to participants is another important feature of effective training programs. Employees who comprehend the potential consequences of noncompliance, including data breaches and compromised patient safety, are more inclined to adopt secure practices (“threat impact assessment” according to the skill model in the present training design). The articulation of the reasons underlying particular security measures ensures that employees not only adhere to the prescribed procedures blindly (if at all) but also discern their significance (“tactic justification” according to the skill model in the present training design). In the long run, this can foster a culture of accountability and vigilance within the organization by promoting consistent and deliberate practice of safe behaviors.

Our empirical findings emphasize that a motivational framing of information security training should align with employees' professional values to maximize its impact. In the context of health care, patient protection was identified as a core value that can demonstrably be addressed through appropriate framing strategies. In other professional fields, formulating an adequate motivational framing necessitates a thorough understanding of the professional values advocated by the respective workforce.

Another recommendation is to introduce the topic of information security early in a professional’s development. This can be done through customized, easily accessible videos, such as those used in this study, or even through playful formats. Addressing this topic during the initial phases of formal vocational education can foster the understanding that information security is an integral part of responsible health care work. Initial vocational education provides more time for reflection on the compatibility of professional values and the protection of sensitive data than short-term pedagogical interventions that accompany daily professional routines and must therefore rely on short framing messages.

From an evaluative perspective, the study underscores the importance of accounting for baseline skills, particularly in light of ceiling effects, where participants with higher pre-training skill levels tend to show less growth. This underlines the need for adaptive and personalized training interventions that can optimize learning outcomes across various starting points [71]. By assessing skill levels across a range of skill sets prior to an intervention, organizations can more effectively target their training efforts to focus on areas where improvement is truly needed. Resources can be allocated to address individual skill gaps rather than providing redundant and one-size-fits-all training to the entire workforce. In the long run, this allows each employee to experience individual skill development throughout the training process, but also facilitates meaningful evaluation of the training program.

### Limitations and Future Research

The study has several limitations that must be acknowledged. First, the investigation was conducted in German university hospitals with a sample of 130 health care professionals, which restricts the generalizability of the findings to other national, cultural, and organizational contexts. While patient protection served as a powerful motivator in the investigated health care context, other professional fields (eg, finance, public administration, and manufacturing) may be guided by entirely different core values, such as diligence, transparency, or efficiency. Further efforts are therefore required to develop problem-oriented, context-sensitive, and profession-specific training content for other occupational fields, as information security demands and role-related responsibilities differ substantially across sectors. In particular, the successful design of motivational framing strategies in these sectors requires a deep understanding of their respective professional ethos.

Second, the study examines short-term effects on skill acquisition, leaving open the question of long-term retention. Future research should focus on the long-term effects of information security interventions, ideally by using follow-up assessments weeks or months after the initial training. This would allow researchers to determine whether the training effects are stable, fade over time, or possibly even increase through continued application in practice.

Another limitation concerns potential self-selection bias. As participation in the study was voluntary and recruitment was conducted via internal channels, such as newsletters and hospital intranet systems, individuals with a stronger interest in information security, greater intrinsic motivation, or a higher sense of professional responsibility were likely to be overrepresented. This may have led to an overestimation of intervention effects. It certainly helps to explain the relatively high baseline skill levels observed across the sample. Participants with advanced prior knowledge may be overrepresented compared to the general hospital workforce. Incorporating random sampling procedures or mandatory participation (in addition to the reported random assignment to framing conditions) could help to ensure a more representative selection of participants.

Results from exploratory subgroup comparisons for the generally beneficial other-oriented framing condition should encourage future investigations into moderator variables that capture individual features of health care professionals. In this study, a grouping approach to contrast job profiles that typically involve high versus low personal contact with patients (physicians and nurses versus administrative staff) provides preliminary support of the assumption that individual interaction frequency with patients might further enhance the impact of patient-oriented appeals. However, this pattern should be interpreted with caution, as the grouping by job profiles offers only a rough approximation of the psychological conditions that may shape responsiveness to value-based framings. Though commonly used to classify professional functions, job profiles often conceal variability within these profiles. For instance, not all physicians are equally involved in direct patient interaction, while certain administrative staff, such as case managers or receptionists, may routinely interact with patients. To specify features that further enhance or reduce the effectiveness of other-oriented framings in the health care sector, future research should therefore consider direct and controllable measures of individual-level moderators. This approach may also include individual assessments of empathy, moral sensitivity, or motivational regulation styles [72,73]. On a contextual level, variables such as organizational culture or leadership commitment to cybersecurity might also be considered as moderating the perceived relevance of information-secure behavior, engagement with training content, or responsiveness to different motivational framing strategies [74].

Finally, this study has put forth several didactical arguments to substantiate that demonstrable skill gains occur because instructional design elements and, in particular, motivational framing strategies encourage deeper engagement with the training content. Studies in other educational contexts have already established the mediating role of learning-process characteristics such as cognitive elaboration and engagement [75]. For information security training in health care settings, statistical evidence of the mediated learning path is still pending.

### Conclusions

The increasing prevalence of cyberattacks and data breaches, particularly in the health care sector, underscores the need to enhance the information security skills of health care professionals through SETA programs. This experimental study highlights the effectiveness of customized security training videos with strategically framed motivational prompts in improving related skills, as assessed by elaborated SJTs. A key aspect of promoting skill acquisition of professionals is to elucidate that compliance with security policies can be fully compatible with their core professional values and that mastering information secure workplace behaviors contributes to enacting these values.

At least in the health care sector studied, where information security breaches have been documented to jeopardize patient safety, this alignment appears to be beneficial. By establishing a link between internalized professional responsibilities to protect patients and methods to ensure the confidentiality, availability, and integrity of sensitive patient data, employees develop a deeper commitment to security policies and to acquiring the necessary skills. In the long run, motivational framing strategies in information security training can become an important tenet in building organizational resilience to cyberattacks. They can not only raise awareness of security-related issues in day-to-day operations but also cultivate the deliberate and regular use of information-secure behaviors.

## Appendix

Multimedia Appendix 1 CONSORT‐eHEALTH checklist (V 1.6.1).

## Abbreviations

ANCOVA: analysis of covariance
CONSORT: Consolidated Standards of Reporting Trials
FBM: Fogg Behavior Model
PMT: protection motivation theory
SETA: Security Education, Training, and Awareness
SJT: situational judgment test

## Data Availability

The datasets generated or analyzed during this study are available from the corresponding author on reasonable request.
